# Systemic Metabolic Rewiring in a Mouse Model of Left Ventricular Hypertrophy

**DOI:** 10.3390/ijms262010111

**Published:** 2025-10-17

**Authors:** Alexandra V. Schmidt, Tharika Thambidurai, Olivia D’Annibale, Sivakama S. Bharathi, Tim Wood, Eric S. Goetzman, Julian E. Stelzer

**Affiliations:** 1 Department of Physiology and Biophysics, School of Medicine, Case Western Reserve University, Cleveland, OH 44106, USA; avs57@case.edu (A.V.S.);; 2 Biochemical Genetics Laboratory, Children’s Hospital of Colorado Anschutz Medical Campus, Aurora, CO 80045, USA; 3 Section of Clinical and Genetics Metabolism, Department of Pediatrics, University of Colorado School of Medicine, Aurora, CO 80045, USA; 4 Division of Human Genetics, Department of Pediatrics, Cincinnati Children’s Hospital Medical Center, Cincinnati, OH 45229, USA; 5 Department of Pediatrics, School of Medicine, University of Pittsburgh, Pittsburgh, PA 15224, USA

**Keywords:** left ventricular hypertrophy, systemic metabolism, cardiac lipotoxicity, cardiac myosin-binding protein C, long-chain fatty acid

## Abstract

Left ventricular hypertrophy (LVH) refers to the pathological thickening of the myocardial wall and is strongly associated with several adverse cardiac outcomes and sudden cardiac death. While the biomechanical drivers of LVH are well established, growing evidence points to a critical role for cardiac and systemic metabolism in modulating hypertrophic remodeling and disease pathogenesis. Despite the efficiency of fatty acid oxidation (FAO), LVH hearts preferentially increase glucose uptake and catabolism to drive glycolysis and oxidative phosphorylation (OXPHOS). The development of therapies to increase and enhance LFCA FAO is underway, with promising results. However, the mechanisms of systemic metabolic states and LCFA dynamics in the context of cardiac hypertrophy remain incompletely understood. Further, it is unknown to what extent cardiac metabolism is influenced by whole-body energy balance and lipid profiles, despite the common occurrence of lipotoxicity in LVH. In this study, we measured whole-body and cellular respiration along with analysis of lipid and glycogen stores in a mouse model of LVH. We found that loss of the cardiac-specific gene, *myosin-binding protein C3* (*Mybpc3*), resulted in depletion of adipose tissue, decreased mitochondrial function in skeletal muscle, increased lipid accumulation in both the heart and liver, and loss of whole-body metabolic flux. We found that supplementation of exogenous LCFAs boosted LVH mitochondrial function and reversed cardiac lipid accumulation but did not fully reverse the hypertrophied heart nor systemic metabolic phenotypes. This study indicates that the LVH phenotype caused systemic metabolic rewiring in *Mybpc3^−/−^* mice and that exogenous LCFA supplementation boosted mitochondrial function in both cardiac and skeletal muscle.

## 1. Introduction

Left ventricular hypertrophy (LVH) is a structural and functional adaptation of the heart in response to increased hemodynamic pressure or volumetric stress caused by increased pressure overload, afterload, or inherited cardiomyopathies. Myocardial wall thickening counteracts increased filling pressures, but oftentimes results in maladaptive remodeling that affects systolic and diastolic function [1]. LVH is commonly associated with comorbidities, such as hypertension, obesity, chronic kidney disease, and diabetes [2,3], and although it is initially compensatory, sustained LVH results in elevated risks of several adverse cardiac outcomes and sudden cardiac death [2]. While the biomechanical drivers of LVH are well established, growing evidence points to a critical role for cardiac and systemic metabolism in modulating hypertrophic remodeling and disease pathogenesis [4]. Pathogenic variants in the cardiac-specific gene, *Mybpc3*, are a leading cause of hypertrophic cardiomyopathies [5]. *Mybpc3*’s product, cardiac myosin-binding protein C (cMyBP-C), is found on the thick filament of cardiac sarcomeres and is thought to function as a major regulator of cardiac contraction. Genetic ablation of *Mybpc3* causes myocardial disarray, impaired in vivo cardiac function, and reduces myofilament Ca^2+^ sensitivity, leading to cardiac fibrosis and hypertrophy in mouse models [6,7].

Long-chain fatty acids (LCFAs) represent the primary energy substrate for the adult heart under normal physiological conditions, despite the heart’s ability to catabolize many different substrates to fuel oxidative phosphorylation (OXPHOS) in mitochondria. However, during pathological hypertrophy, cardiac substrate utilization shifts away from fatty acid oxidation (FAO)-derived OXPHOS toward glucose-derived OXPHOS [4,8]. Since the heart requires a tremendous amount of energy in the form of ATP to support the demands of the cardiac cycle, it has been suggested that this metabolic rewiring compromises the efficiency of myocardial bioenergetics [8,9]. It is thought that this metabolic rewiring is somewhat inflexible due to the hypoxic conditions that arise in a hypertrophic muscle [10].

Recent studies [11,12] have explored the therapeutic potential of restoring or enhancing LCFA oxidation to improve cardiac metabolic flux and attenuate hypertrophy. Interventions targeting LCFA transport, mitochondrial uptake, or increasing FAO gene expression with the use of peroxisome proliferator-activated receptor (PPAR) agonist drugs have shown promise in both pre-clinical studies and/or clinical trials [13,14,15,16]. Yet, the mechanisms of systemic metabolic states and LCFA dynamics in the context of cardiac hypertrophy remain incompletely understood. Further, it is unknown to what extent cardiac metabolism is influenced by whole-body energy balance and lipid profiles, despite the common occurrence of lipotoxicity (accumulation of fatty acid metabolites and intermediates stored in lipid particles) in hypertrophied myocardium [17].

With the growing interest in leveraging metabolism in treating LVH and a wide range of other cardiovascular diseases (CVDs), a better understanding of the systemic adaptations caused by LVH is necessary. Here, we show that mice with LVH due to the absence of the cardiac-specific regulatory protein, cMyBP-C, display a shift in systemic metabolism and depleted energy stores, independent of decreased cardiac output. Our findings suggest that a systemic shift in metabolism is due to the metabolic inflexibility of the heart, requiring other highly metabolic organs, such as the liver and skeletal muscle, to compensate for altered cardiac substrate handling.

## 2. Results

### 2.1. Mybpc3^−/−^ Mice Have Altered Respiration Compared to Wild-Type Mice

**Figure 1:**
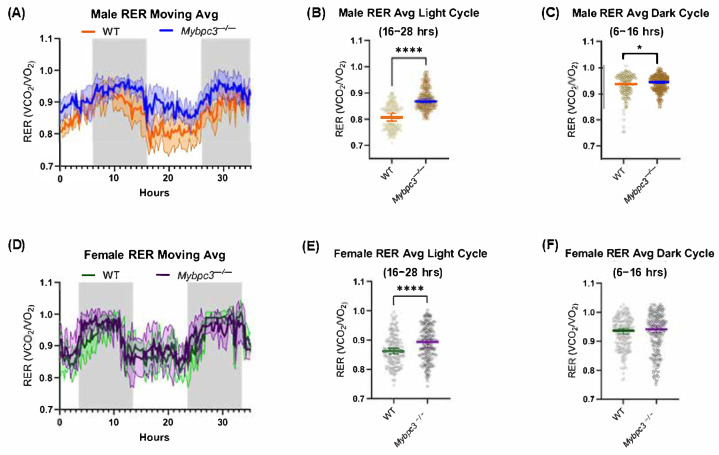
(**A**) IDC data from WT (*N* = 5) and *Mybpc3^−/−^* (*N* = 7) males are represented as a moving average of RER. The average RER of *Mybpc3^−/−^* males (AUC = 8.473 ± 0.08646) was higher than WT males (AUC = 6.553 ± 0.09763) over the course of 48 h. Error bars (shaded region) represent SD. (**B**) Individual RER values from each male mouse cohort were restricted to one light cycle (16–28 h) for cycle-specific statistical analysis. *Mybpc3^−/−^* males (mean = 0.8773) had significantly higher RER readings compared to WT males (mean = 0.8079) during the light cycle (unpaired *t*-test; *p* < 0.0001). Error bars represent SEM. (**C**) Individual RER values from each male mouse cohort were restricted to one dark cycle (6–16 h) for cycle-specific statistical analysis. *Mybpc3^−/−^* males (mean = 0.9398) had significantly higher RER values during the dark cycle compared to WT males (mean = 0.9305; unpaired *t*-test; *p* = 0.0137). Error bars represent SEM. (**D**) Female IDC data from WT (*N* = 5) and *Mybpc3^−/−^* (*N* = 5) mice are represented as a moving average of RER. The average RER of *Mybpc3^−/−^* females (AUC = 7.911 ± 0.1097) was similar to WT females (AUC = 7.563 ± 0.09611) over the course of 48 h. Error bars represent SD. (**E**) Female *Mybpc3^−/−^* mice (mean = 0.8886) had significantly higher RER readings compared to WT females (mean = 0.8654) during the light cycle (unpaired *t*-test; *p* < 0.0001). Error bars represent SEM. (**F**) There was no significant difference in mean RER values between *Mybpc3^−/−^* vs. WT females during the dark cycle (unpaired *t*-test; *p* = 0.4448). Error bars represent SEM. * = *p* < 0.05; **** = *p* < 0.0001.

Indirect calorimetry was used to determine whether whole-body respiration was different between *Mybpc3* knockout (*Mybpc3^−/−^*) and wild-type (WT) mice. The respiratory exchange ratio (RER), calculated as the ratio of CO_2_ exhaled over O_2_ consumed, is an indicator of whole-body fuel selection, with a ratio of 1.0 indicating pure carbohydrate oxidation and 0.7 indicating pure fat oxidation. Normal mice preferentially oxidize carbohydrates during the dark cycle and shift toward fat oxidation while resting during the light cycle. A moving average of RER in male *Mybpc3^−/−^* mice indicated that they had elevated RER values during the 48 h experiment (Figure 1A). This was most striking during the light cycle, indicating that *Mybpc3^−/−^* mice failed to shift their metabolism toward fat oxidation when less active during the day (Figure 1B). Similar results were observed in female *Mybpc3^−/−^* mice. There was no significant difference in food intake between *Mybpc3^−/−^* and WT mice of both sexes (Figure S1).

To gain further insight into the resting metabolic rate of *Mybpc3^−/−^* mice compared to controls, O_2_ consumption was compared between *Mybpc3^−/−^* and their WT counterparts. A two-way analysis of covariance (ANCOVA) test was performed with genotype and sex as fixed factors, and body mass as a covariate [18]. During the light cycle, there were no significant differences in O_2_ consumption between the two genotypes (F = 0.0862, *p* = 0.7712) for either sex (F = 0.1400, *p* = 0711). There were also no significant differences in O_2_ consumption for each genotype (F = 0.0292, *p* = 0.8655) during the dark cycle in either sex (F = 0.6417, *p* = 0.4299) (Table 1). This suggested that *Mybpc3^−/−^* had a more rigid metabolic flux and were more reliant on glucose oxidation at the whole-body level without influence from differences in O_2_ consumption.

### 2.2. Mybpc3^−/−^ Mice Have Altered Substrate Stores Compared to Wild-Type Mice

**Figure 2:**
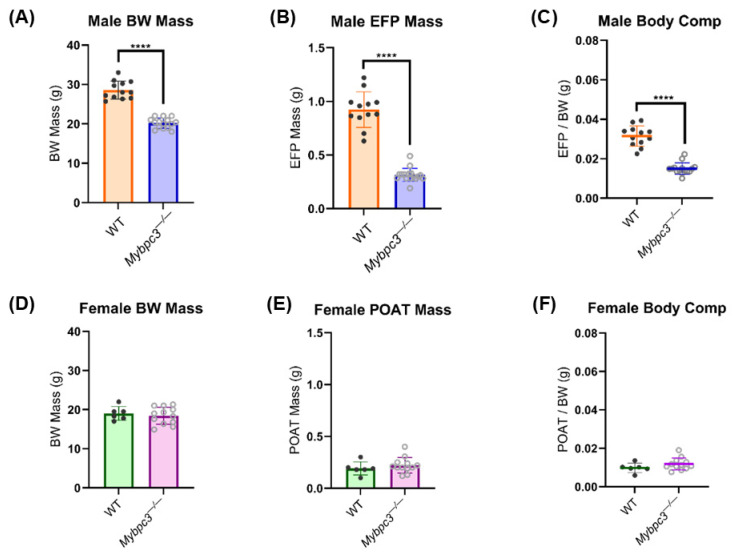
(**A**) Male *Mybpc3^−/−^* mice (*N* = 17) had significantly lower BW mass compared to age-matched WT males (*N* = 12; unpaired *t*-test; *p* < 0.0001). (**B**) Male *Mybpc3^−/−^* mice *(N* = 17) had significantly lower EFP mass compared to age-matched male WT mice (*N* = 12; unpaired *t*-test; *p* < 0.0001). (**C**) Male EFP weights were normalized to BW to calculate an EFP/BW ratio to serve as an estimation of body composition. An unpaired *t*-test showed that male *Mybpc3^−/−^* mice had a significantly lower EFP/BW ratio compared to WT males (unpaired *t*-test; *p* < 0.0001). (**D**) There was no difference in female *Mybpc3^−/−^* mice (*N* = 12) BW mass compared to age-matched WT females (*N* = 6; unpaired *t*-test; *p* = 0.5803). (**E**) There was no significant difference between female *Mybpc^−^^/−^* mice (*N* = 12) POAT mass compared to age-matched female WT mice (*N* = 6) POAT mass (unpaired *t*-test; *p* = 0.4091). (**F**) Female POAT weights were normalized to BW to calculate a POAT/BW ratio to serve as an estimation of body composition. An unpaired *t*-test showed that female *Mybpc3^−/−^* and female WT mice had no significant difference in the POAT/BW ratio (unpaired *t*-test; *p* = 0.4091). **** = *p* < 0.0001. All error bars represent SD.

To estimate body composition, the gonadal fat pads of *Mybpc3^−/−^* and WT male and female mice were isolated, weighed, and normalized to body weight (BW) [19]. Both the average total body weight and average epididymal fat pad (EFP) weight were significantly lower in *Mybpc3^−/−^* male mice compared to WT counterparts (Figure 2A,B). When EFP weight was normalized to total BW for each mouse, male *Mybpc3^−/−^* mice had a two-fold decrease in the EFP/body weight ratio compared to WT mice, indicating that *Mybpc3^−/−^* mice had significantly less gonadal adiposity compared to WT mice (Figure 2C). However, there was no difference in total BW (Figure 2D), periovarian adipose tissue (POAT) (Figure 2E), nor POAT/BW measurements between *Mybpc3^−/−^* and WT females (Figure 2F).

**Figure 3:**
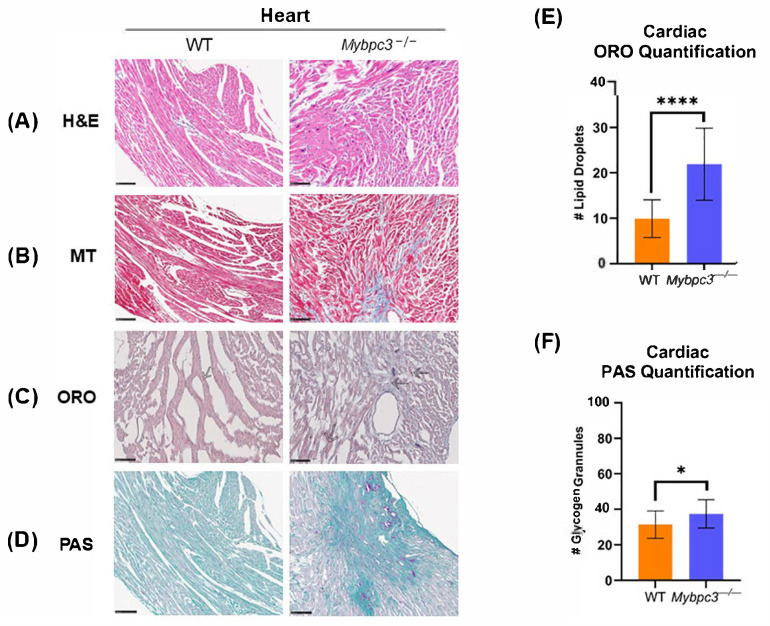
Histological analysis was performed in cardiac tissue collected from male *Mybpc3^−/−^* and WT mice. (**A**) H&E staining shows that *Mybpc3^−/−^* mice have differences in cardiomyocyte size and organization. (**B**) MT staining shows that *Mybpc3^−/−^* mice have clear, large regions of collagen deposition. (**C**) ORO staining shows that *Mybpc3^−/−^* cardiac tissue has an accumulation of large lipid droplets (as indicated by arrows). (**D**) PAS staining indicated glycogen-positive cardiomyocytes in both WT and *Mybpc3^−/−^* cardiac tissues. Scale bar represents 100 μM; *N* = 3 for each group. (**E**) The number of lipid droplets per 600 μM in *Mybpc3^−/−^* mouse (*N* = 15) cardiac tissue was significantly higher compared to WT mice (*N* = 15; unpaired *t*-test; *p* < 0.0001). (**F**) The number of glycogen granules per 600 μM was significantly higher in *Mybpc3^−/−^* (*N* = 15) cardiac tissue compared to WT mice (*N* = 15; unpaired *t*-test; *p* = 0.0460). * = *p* < 0.05; **** = *p* < 0.0001. All error bars represent SD.

**Figure 4:**
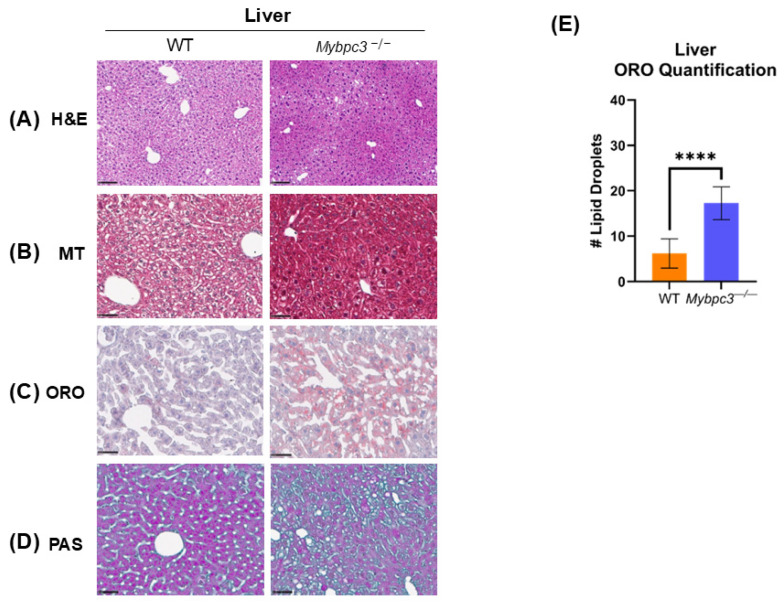
Histological analysis was performed in liver tissue collected from male *Mybpc3^−/−^* and WT mice. (**A**) H&E staining shows no clear differences in hepatocyte size or organization. (**B**) MT staining shows that *Mybpc3^−/−^* mice have clear, large regions of collagen deposition. (**C**) ORO staining shows that *Mybpc3^−/−^* hepatic tissue has an accumulation of large lipid droplets. (**D**) PAS staining indicated glycogen-positive cardiomyocytes in both WT and *Mybpc3^−/−^* cardiac tissues. Scale bar represents 100 μM; *N* = 3 for each group. (**E**) *Mybpc3^−/−^* mice (*N* = 15) had significantly more lipid droplets in the liver per 600 μM compared to WT mice (*N* = 15; *p* < 0.0001). **** = *p* < 0.0001. Error bars represent SD.

Given the increased RER and lack of adipose tissue in male *Mybpc3^−/−^* mice, we wanted to investigate whether *Mybpc3^−/−^* mice had access to the fatty acids, stored as triglycerides in lipid droplets, that drive FAO OXPHOS in oxidative tissues. A histology study with Oil Red O (ORO) staining was performed to measure lipid droplet accumulation in cardiac (Figure 3) and liver (Figure 4) tissue collected from *Mybpc3^−/−^* and WT mice. WT mice had significantly less lipid droplet accumulation in cardiac tissue compared to *Mybpc3^−/−^* tissue (Figure 3E), suggesting that this model of LVH had lipotoxicity, consistent with clinical data and other animal models [20,21,22]. *Mybpc3^−/−^* mice also had increased lipid droplet accumulation in hepatic tissue (Figure 4E), suggesting a fatty liver phenotype [23]. Quantitative analysis of Periodic Acid–Schiff (PAS) staining showed that *Mybpc3^−/−^* mice had significantly more glycogen-positive cardiomyocytes compared to WT mice (Figure 3F). Whole-slide scans of liver PAS stains suggested fewer glycogen-rich regions in *Mybpc3^−/−^* livers compared to WT livers (Figure S2).

Hematoxylin and Eosin (H&E) and Masson’s Trichrome (MT) staining were also performed in these tissues to assess tissue architecture, hypertrophy, and tissue damage (Figure 3 and Figure 4). H&E stains of cardiac tissue showed dysmorphic, unorganized cardiomyocytes in the *Mybpc3^−/−^* mice due to the LVH phenotype (Figure 3A). MT staining showed collagen deposition and development of fibrotic tissue in *Mybpc3^−/−^* males, as expected (Figure 3B). H&E and MT staining did not uncover any tissue damage in the *Mybpc3^−/−^* liver (Figure 4A), despite lipid droplet accumulation (Figure 4C,E) and potential reduction in glycogen stores (Figure 4D).

### 2.3. Circulating Metabolites Differ Between Mybpc3^−/−^ and Wild-Type Males

**Figure 5:**
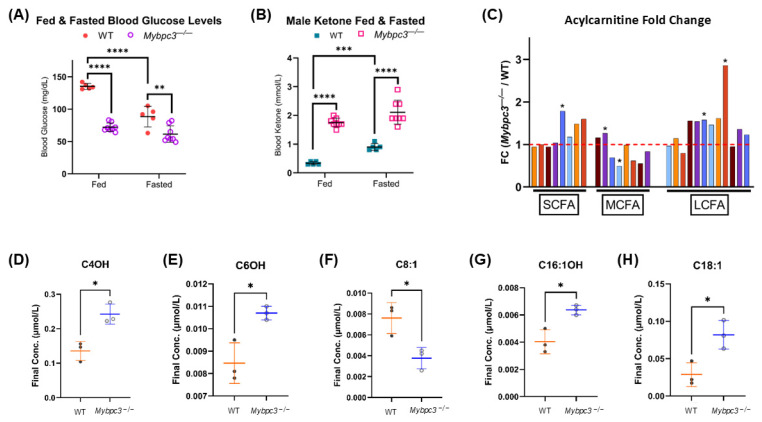
(**A**) Two-way ANOVA mixed-effects analysis was performed to determine the effect of fasting on blood glucose levels in *Mybpc3^−/−^* (*N* = 8) vs. WT (*N* = 8) male mice. Overall, fasting only decreased blood glucose levels in WT mice. Fed WT mice had significantly higher blood glucose levels compared to *Mybpc3^−/−^* mice (2-way ANOVA; *p* < 0.0001). (**B**) Male *Mybpc3^−/−^* had significantly higher blood ketone levels compared to WT mice in both a fed (2-way ANOVA; *p* < 0.00001) and fasted (2-way ANOVA; *p* < 0.00001) state. WT mice had a significant increase in blood ketone levels in a fasted state compared to a fed state (2-way ANOVA; *p* < 0.0001). (**C**) Fold change (FC) was calculated for each acyl-CoA species by dividing the mean *Mybpc3^−/−^* concentration by the mean WT concentration. Values over 1.0 indicate an increase in *Mybpc3^−/−^* mice; values below 1.0 indicate a decrease in *Mybpc3^−/−^* mice. *p*-values were calculated using the mean of each genotype to determine significant increases/decreases. (**D**–**H**) All acylcarnitine chain lengths with significant differences in mean concentrations are shown. * = *p* < 0.05; ** = *p* < 0.005; *** = *p* < 0.0005; **** = *p* < 0.0001. Error bars represent SD.

Since both light and dark cycle RER measurements and body composition estimates were significantly different in males, but not females, we focused further metabolic flux experiments on male mice. To determine whether *Mybpc3^−/−^* mice had altered circulating metabolites potentially causing altered substrate stores in the heart and liver, blood glucose and ketone levels were measured before and after a 12 h fasting challenge. Blood glucose levels in WT animals significantly decreased post-fast, as expected. *Mybpc3^−/−^* male glucose levels were significantly lower than WT mice before and after the 12 h challenge, suggesting inflexibility of carbohydrate metabolism (Figure 5A). *Mybpc3^−/−^* males had significantly higher blood ketone levels before and after the fasting challenge (Figure 5B). Mean blood ketone levels almost tripled from 0.35 mmol/L in a fed state to 0.90 mmol/L in a fasted state in WT mice. However, *Mybpc3^−/−^* mice had mean blood ketone levels around 2.0 mmol/L in both a fed and fasted state, reaching blood ketone levels typically observed in mouse models of ketoacidosis [24,25].

Circulating glucose and ketone data indicated that *Mybpc3^−/−^* mice were in metabolic duress at the whole-body level. To better understand how this affected the metabolism of fatty acids, an acylcarnitine profiling study was performed in blood serum. The acylcarnitine profile is a well-known biomarker of FAO flux in vivo. We found that the biggest difference in acylcarnitines was a significant, three-fold increase in C18:1 carnitine in *Mybpc3^−/−^* mice (Figure 5H). In fact, there was a slight elevation in most long-chain carnitine species (Figure 5C), suggesting that *Mybpc3^−/−^* mice were not only more reliant on glucose catabolism and oxidation for energy production but that FAO-linked OXPHOS was inefficiently using available LCFAs after first-pass FAO.

**Figure 6:**
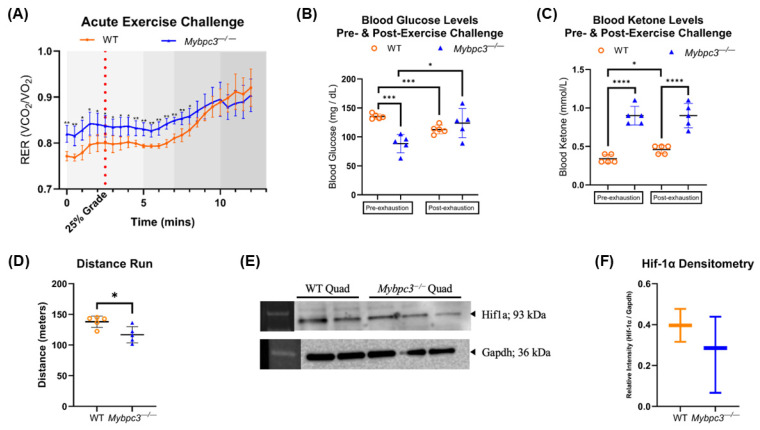
(**A**) RER values within each genotype were averaged and plotted as a moving RER average against time. Unpaired *t*-tests to compare WT and *Mybpc3^−/−^* mean at each time point (X-axis) were performed. (**B**) Blood glucose levels measured pre- and post-exhaustion were plotted for *Mybpc3^−/−^* male mice (*N* = 5) and WT mice (*N* = 5). A paired *t*-test showed that WT mice had a significant decrease in blood glucose levels post-exhaustion (*p* = 0.0009). A paired *t*-test showed that *Mybpc3^−/−^* mice (*N* = 5) had a significant increase in blood glucose levels post-exhaustion (*p* = 0.0429). An unpaired *t*-test showed that *Mybpc3^−/−^* mice had significantly lower blood glucose levels than WT mice pre-exhaustion (*p* = 0.0002). (**C**) Blood ketone levels measured pre- and post-exhaustion were plotted for *Mybpc3^−/−^* male mice (*N* = 5) and WT mice (*N* = 5). *Mybpc3^−/−^* mice had significantly higher blood ketone levels pre-exhaustion compared to WT mice (*p* < 0.0001). *Mybpc3^−/−^* mice had significantly lower blood ketone levels compared to WT mice post-exhaustion (*p* < 0.0001). WT mice had significantly higher blood ketone levels post-exercise challenge (*p* = 0.0327). (**D**) *Mybpc3^−/−^* mice ran a significantly shorter distance compared to WT mice (unpaired *t*-test; *p* = 0.0192). (**E**) Immunoblot data showed that Hif-1α was present at low levels in both *Mybpc3^−/−^* and WT quadriceps. Anti-Gapdh was used as a control. (**F**) Densitometric analysis of Hif-1α band intensity normalized to Gapdh band intensity showed no significant difference in protein levels (*p* = 0.3976) between *Mybpc3^−/−^* mice (*N* = 3) vs. WT mice (*N* = 2). * = *p* < 0.05; ** = *p* < 0.005; *** = *p* < 0.0001; **** = *p* < 0.00001. All error bars represent SD.

The elevated ketones shown earlier in *Mybpc3^−/−^* mice indicated higher liver long-chain FAO, as FAO is the source of mitochondrial acetyl-CoA during ketogenesis. Yet, the serum acylcarnitine profile indicated inefficient FAO at the whole-body level. It has been shown that the serum acylcarnitine profile primarily reflects cardiac muscle FAO, and secondarily skeletal muscle FAO [26]. The muscle is also the major contributing factor to RER measurements at rest and during exercise [27], which is further influenced by muscle fiber type [28]. To determine whether the apparent metabolic inflexibility in *Mybpc3^−/−^* mice reflects changes in muscle physiology, exercise-naïve mice were given an acute exercise challenge. IDC was used to monitor RER during the initial 15 min of treadmill running, with glucose and ketone body levels measured pre- and post-exhaustion. *Mybpc3^−/−^* mice had significantly higher RER and blood ketone levels pre-exhaustion, as described earlier. WT mice ran significantly further than *Mybpc3^−/−^* mice, indicating that LVH caused an exercise intolerance phenotype. *Mybpc3^−/−^* mice did have similar RER to WT mice as the exercise challenge progressed past the “aerobic” portion (Figure 6A). However, *Mybpc3^−/−^* mice post-exhaustion had significantly higher glucose levels compared to WT mice (Figure 6B). Ketone levels were equal pre- and post-exhaustion in both *Mybpc3^−/−^* and WT mice. Although, *Mybpc3^−/−^* mice did have significantly higher blood ketone levels pre- and post-exhaustion (Figure 6C). Hypoxia-inducible factor 1-alpha (Hif-1α) is a known regulator of glycolysis and has been shown to be elevated in the *Mybpc3^−/−^* heart due to the LVH phenotype [29]. To test whether a similar mechanism extends to the skeletal muscle, immunoblotting was performed to detect Hif-1α. Densitometric analysis indicated no differences in Hif-1α protein levels between the two genotypes (Figure 6E,F).

### 2.4. Mybpc3^−/−^ Males Have Decreased OXPHOS Capacity in the Heart and Skeletal Muscle Compared to Wild-Type Males

**Figure 7:**
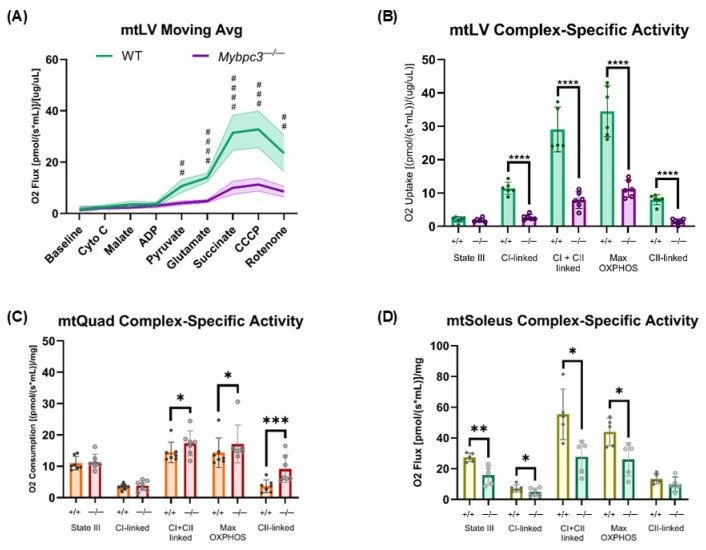
(**A**) The mean respiration of WT mtLV (*N* = 6) and *Mybpc3^−/−^* mtLV (*N* = 6) is represented as a moving average in response to acute treatment of various metabolic substrates. AUC analysis showed that WT mtLV had an AUC value of 111.20 ± 8.23 compared to the mtLV *Mybpc3^−/−^* AUC value of 42.48 ± 3.08. Unpaired *t*-test analysis showed that *Mybpc3^−/−^* mtLVs had significantly lower mitochondrial respiration in response to pyruvate (Q = 0.000111), glutamate (Q < 0.000001), succinate (Q = 0.000090), CCCP (*p* = 0.000090), and rotenone (Q = 0.000632) treatment. (**B**) Complex-specific activity was calculated in WT (*N* = 6) and *Mybpc3^−/−^* (*N* = 6) mtLV. *Mybpc3^−/−^* mtLV had significantly reduced CI-linked (*p* < 0.0001), CI + CII-linked (*p* < 0.0001), max OXPHOS (*p* < 0.0001), and CII-linked (*p* < 0.0001) compared to WT mtLV. (**C**) Complex-specific activity was calculated from mean oxygen consumption in mtQuad isolated from WT mice (*N* = 7) and *Mybpc3^−/−^* mice (*N* = 7) quadriceps. Unpaired *t*-tests showed that mtQuad from *Mybpc3^−/−^* mice was significantly increased in CI + CII-linked (*p* = 0.0111), max OXPHOS (*p* = 0.0469), and CII-linked (*p* = 0.0030) compared to WT mtQuad. (**D**) Complex-specific activity was calculated from mean oxygen consumption in mtSoleus isolated from WT mice (*N* = 5) and *Mybpc3^−/−^* mice (*N* = 5). Unpaired *t*-tests showed that mtSoleus from *Mybpc3^−/−^* mice had significantly decreased State III (*p* = 0.0060), CI-linked (0.0430), CI + CII-linked (*p* = 0.0140), and max OXPHOS (*p* = 0.0222) compared to WT mtSoleus. Error bars represent SD. ## = Q < 0.001; ### = Q < 0.0001; #### = Q > 0.00001. * = *p* < 0.05; ** = *p* < 0.005; *** = *p* < 0.0005; **** = *p* < 0.00001.

Mitochondria isolated from the LV (mtLV) of *Mybpc3^−/−^* mice have significantly decreased oxygen consumption compared to WT mtLV with the supplementation of oxidative substrates pyruvate, glutamate, and succinate (Figure 7A). Both NADH-dependent complex CI, FADH2-activated complex CII, and maximal OXPHOS capacity were significantly reduced in *Mybpc3^−/−^* mtLV (Figure 7B). However, State III respiration was not significantly different. This suggested that the proton gradient, which is in essence potential energy within the inner mitochondrial membrane (IMM) in the form of H^+^, could be disrupted in *Mybpc3^−/−^* mouse mtLV.

Given that we found that systemic metabolism was significantly different between *Mybpc3^−/−^* and WT males, it is possible that skeletal muscle mitochondria could have differences in metabolism indirectly caused by LVH. We found that mitochondria isolated from the quadriceps (mtQuad), which is a glycolytic, fast-twitch muscle, had no differences in State III or CI-linked activity in *Mybpc3^−/−^* activity compared to WT mtQuad. However, CII-linked, CI- + CII-linked (likely due to increased CII activity), and maximal OXPHOS were all significantly higher in *Mybpc3^−/−^* mtQuad (Figure 7C). Similar to mtLV, mitochondria isolated from slow-twitch, FAO-dependent soleus tissue (mtSoleus) in *Mybpc3^−/−^* mice had significantly lower CI-linked, CI- + CII-linked, and maximal OXPHOS capacity compared to WT mtSoleus (Figure 7D).

### 2.5. Long-Chain Fatty Acid Supplementation Ameliorates Decreased OXPHOS Capacity in Mybpc3^−/−^ Mouse Skeletal Muscles

**Figure 8:**
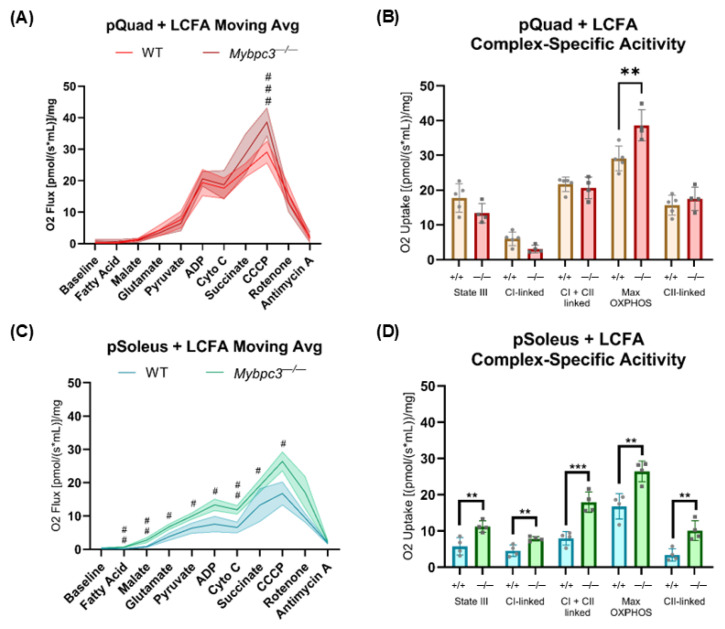
(**A**) Metabolic flux is represented as oxygen flux in response to acute treatment of various substrates in permeabilized quadriceps muscle fibers (pQuad) collected from *Mybpc3^−/−^* mice (*N* = 4) and WT mice (*N* = 5). AUC analysis indicated that WT and *Mybpc3^−/−^* pQuad had similar area values, with 119.5 ± 5.664 and 132.5 ± 7.173, respectively. *Mybpc3^−/−^* pQuad had a significantly higher response to CCCP (Q < 0.0001) compared to WT mtQuad. (**B**) State III, maximum OXPHOS capacity, and complex-specific activity were calculated for pQuad collected from *Mybpc3^−/−^* and WT mice with treatment of a long-chain fatty acid (LCFA). *Mybpc3^−/−^* pQuad (*N* = 4) had significantly higher oxygen uptake only at maximal OXPHOS compared to WT pQuad (*N* = 5; *p* = 0.0089). (**C**) Metabolic flux is represented as oxygen flux in response to acute treatment of various substrates, including an LCFA, in permeabilized soleus muscle fibers (pSoleus). AUC analysis showed that the total peak area in *Mybpc3^−/−^* pSoleus was higher than WT pSoleus, with 109.1 ± 4.557 and 65.7 ± 5.007, respectively. *Mybpc3^−/−^* pSoleus samples had significantly higher oxygen influx after fatty acid (Q = 0.0061), malate (Q = 0.0061), glutamate (Q = 0.0085), pyruvate (Q = 0.0091), ADP (Q = 0.0091), cytochrome c (Q = 0.0061), and CCCP (Q = 0.0085) treatment. (**D**) State III, maximum OXPHOS capacity, and complex-specific activity were calculated for pSoleus collected from *Mybpc3^−/−^* and WT mice with treatment of a long-chain fatty acid (LCFA). State III (*p* = 0.0087), CI-linked (*p* = 0.0083), CI- + CII-linked (*p* = 0.0010), maximum OXPHOS (*p* = 0.0052), and CII-linked (*p* = 0.0059) were all significantly increased in *Mybpc3^−/−^* mice (*N* = 4) compared to WT mice (*N* = 4). Error bars represent SD. # = Q < 0.01; ## = Q < 0.001; ### = Q < 0.0001. ** = *p* < 0.005; *** = *p* < 0.0005.

Our finding that there was reduced OXPHOS capacity in a highly metabolic tissue outside the heart was surprising. Moreover, this reduced OXPHOS capacity was specific to tissues that typically rely on FAO-mediated OXPHOS [30]. We hypothesized that exogenous supplementation of a long-chain fatty acid (LCFA), palmitoyl-L-carnitine, would reverse the reduced mitochondrial capacity in mtLV and mtSoleus. To test this, muscle fibers from quadriceps and solei were collected and permeabilized with saponin as previously described [31]. This facilitated LCFA uptake independent of fatty acid transporters. Treatment of permeabilized muscle fibers from quadriceps (pQuad) did not appear to affect FAO-mediated OXPHOS between *Mybpc3^−/−^* and WT pQuad. However, significantly increased CCCP response and max OXPHOS data indicated that maximal OXPHOS capacity was slightly increased in *Mybpc3^−/−^* pQuad after treatment of the tissue fibers with palmitoyl-L-carnitine (Figure 8A,B). Supplementation of palmitoyl-L-carnitine to pSoleus increased oxygen flux in *Mybpc3^−/−^* solei by almost two-fold (Figure 8C). Furthermore, linked complex activities were all significantly increased in *Mybpc3^−/−^* pSolei, with a three-fold increase in both CI + CII-linked OXPHOS and CII-linked OXPHOS (Figure 8D). Maximum OXPHOS capacity was also significantly higher in pSoleus from *Mybpc3^−/−^* mice.

### 2.6. Exogenous Supplementation of LCFAs Bypasses Lipid Storage in Mybpc3^−/−^ Mice

**Figure 9:**
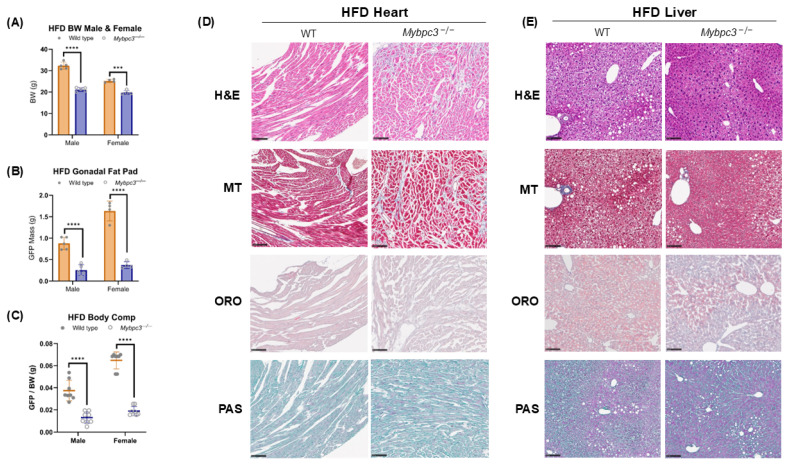
*Mybpc3^−/−^* mice and WT mice of both sexes were fed an HFD for 10 weeks. (**A**) Male *Mybpc3^−/−^* mice (*N* = 5) had a significantly lower body weight compared to WT male mice (*N* = 5; *p* < 0.000001). Female *Mybpc3^−/−^* mice (*N* = 4) had a significantly lower body weight compared to female WT mice (*N* = 4; *p* = 0.000131). (**B**) Male *Mybpc3^−/−^* mice (*N* = 5) had a significantly lower mean GFP (EFP) weight compared to WT male mice (*N* = 5; *p* = 0.000085). Female *Mybpc3^−/−^* mice (*N* = 4) had a significantly lower mean GFP (POAT) weight compared to WT mice (*N* = 4). (**C**) Male EFP weights were normalized to BW to calculate an EFP/BW ratio to serve as an estimation of body composition. Two-way ANOVA analysis showed that both male (*N* = 8) and female (*N* = 8) *Mybpc3^−/−^* mice had a significantly lower GFP/BW ratio compared to WT males (*N* = 8) and females (*N* = 7; F (1, 15) = 37.52; *p* < 0.00001). (**D**) A qualitative histology study was performed with H&E, MT, ORO, and PAS in heart tissue isolated from WT and *Mybpc3^−/−^* male mice fed an HFD for 10 weeks. (**E**) A qualitative histology study was performed with H&E, MT, ORO, and PAS in heart and liver tissue isolated from WT and *Mybpc3^−/−^* male mice fed an HFD for 10 weeks. *** = *p* < 0.0005; **** = *p* < 0.00001. All error bars represent SD.

Since we observed a significant increase in mitochondrial respiration in *Mybpc3^−/−^* soleus tissue, we hypothesized that systemic *Mybpc3^−/−^* metabolism would increase as a result of exogenous LCFA supplementation in the form of a high-fat diet (HFD; >60% kcal from lard). *Mybpc3^−/−^* and WT controls of both sexes were fed an HFD for 10 weeks to test whether exogenous LCFA supplementation could affect the differences in body composition, metabolic deficits, and substrate handling in vivo. Both male and female *Mybpc3^−/−^* mice had significantly lower mean BWs (Figure 9A) and GFP masses (Figure 9B) compared to WT mice. Although both male and female *Mybpc3^−/−^* mice on an HFD had significantly reduced GFP adiposity compared to WT mice (Figure 7C), intracellular lipid stores appeared to change as a result of an HFD. ORO staining of WT cardiac tissue indicated accumulation of large lipid droplets, which were not present in *Mybpc3^−/−^* tissue samples (Figure 9D). Clear, large lipid droplets were visible in H&E, MT, ORO, and PAS stains of WT liver tissues. However, *Mybpc3^−/−^* mice did not appear to have similar clear, large lipid droplets visible in H&E, MT, ORO, or PAS stains (Figure 9E). Both *Mybpc3^−/−^* and WT mice had glycogen-rich and glycogen-depleted regions in hepatic tissue (Figure S5). Despite these differences in lipid accumulation, *Mybpc3^−/−^* on an HFD presented with similar pathogenic changes in tissue architecture and collagen deposition as *Mybpc3^−/−^* mice on a standard lab diet, indicating that the HFD did not rescue LVH pathogenesis (Figure 9D).

### 2.7. A High-Fat Diet Boosts Mybpc3^−/−^ Cardiac Respiration

Experiments to challenge cardiomyocyte and whole-body metabolism were repeated in *Mybpc3^−/−^* males fed an HFD for 10 weeks to assess the impact of altered lipid stores due to exogenous LCFA supplementation. One-way ANCOVA analysis of O_2_ consumption in *Mybpc3^−/−^* males compared to WT males showed no significant effects (F = 0.9538, *p* = 0.3543) (Table 2).

**Figure 10:**
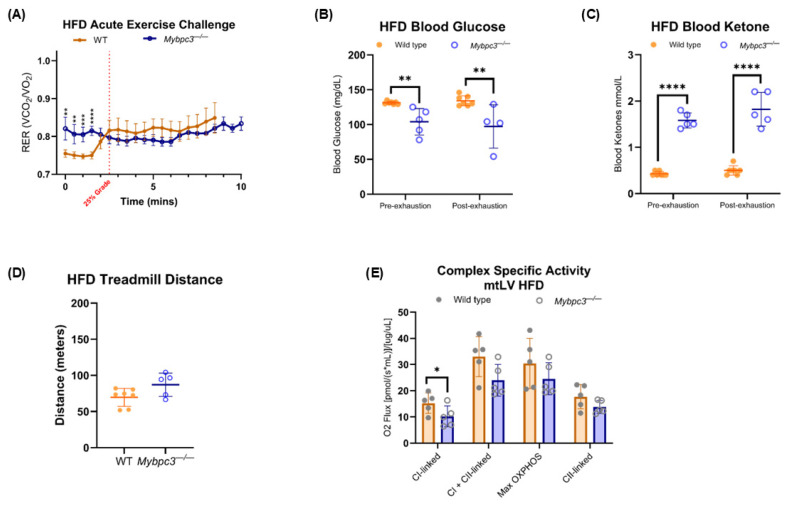
WT and *Mybpc3^−/−^* mice were fed an HFD for 10 weeks. (**A**) Exercise-naïve *Mybpc3^−/−^* and WT mice were subject to an acute exercise challenge, reported as an RER moving average. AUC analysis was performed; WT mice (*N* = 7) had a peak area value of 6.835 ± 0.036, and *Mybpc3^−/−^* mice (*N* = 5) had a peak area value of 8.044 ± 0.023. *Mybpc3^−/−^* mice had a significantly higher RER at the beginning of the exercise challenge (Q = 0.0011; Q = 0.0011; Q = 0.0002; Q < 0.00001). (**B**) Two-way ANOVA analysis indicated that *Mybpc3^−/−^* mice (*N* = 5) had significantly lower blood glucose levels compared to WT mice (*N* = 7), both pre- and post-exercise challenge (F (1, 6) = 39.59; *p* = 0.0008). (**C**) Two-way ANOVA analysis indicated that *Mybpc3^−/−^* mice (*N* = 5) had significantly lower blood ketone levels compared to WT mice (*N* = 7), both pre- and post-exercise challenge. (**D**) There was no significant difference in mean distance run between *Mybpc3^−/−^* vs. WT mice (unpaired *t*-test; *p* = 0.0614). (**E**) Complex-specific activity was measured in mtLV collected from *Mybpc3^−/−^* and WT mouse hearts, represented as an oxygen flux. *Mybpc3^−/−^* mice (*N* = 5) had significantly lower CI-linked activity compared to control counterparts (*N* = 5; *p* = 0.0377). CI + CII-linked (*p* = 0.0966), maximum OXPHOS (*p* = 0.2372), and CII-linked (*p* = 0.2073) oxygen flux was not significantly different between *Mybpc3^−/−^* vs. WT mtLV. * = *p* < 0.05; ** = *p* < 0.005; *** = *p* < 0.0005; **** = *p* < 0.00001. All error bars represent SD.

However, there was no glucose catabolism-driven spike in *Mybpc3^−/−^* RER during an acute exercise challenge, suggesting that highly metabolic tissues were more reliant on FAO OXPHOS instead of glycolysis (Figure 10A). Although *Mybpc3^−/−^* resting RER was significantly higher, WT mice experienced a glucose-driven spike in RER during an acute exercise challenge, as expected and seen in both genotypes on a regular diet (Figure 6A). *Mybpc3^−/−^* males had significantly lower glucose levels, but higher ketone body levels, pre- and post-exercise challenge compared to WT mice, despite supplementation with exogenous LCFAs (Figure 10B,C).

## 3. Discussion

The findings presented in this study indicate that *Mybpc3^−/−^* mice exhibit a notable whole-body shift toward increased reliance on glucose catabolism and have reduced mitochondrial respiration in both cardiac and slow-twitch skeletal muscle. *Mybpc3^−/−^* mice showed significantly decreased oxygen consumption and energy expenditure compared to WT mice. This is consistent with previous studies performed with human myocardium isolated from patients with cardiac hypertrophy, which also report shifts in energy metabolism away from FAO and toward glucose catabolism OXPHOS in cardiac tissue [22,32]. However, the observation that *Mybpc3^−/−^* mice display a reduction in whole-body expenditure warrants further investigation to determine the underlying mechanisms of this apparent systemic shift in bioenergetic preference for glycolysis. Although metabolic phenotypes could be affected by activity levels, our treadmill exercise data (Figure 6A) showed that *Mybpc3^−/−^* mice run for the same duration as WT mice under an identical exercise protocol. Additionally, if the LVH phenotype in *Mybpc3^−/−^* mice was restricting pulmonary function and thus limiting exercise capacity, we would have expected to see significant differences in treadmill performance and time to exhaustion. Since *Mybpc3^−/−^* mice performed similarly to WT mice, we can infer that the altered metabolic phenotype is not primarily driven by decreased exercise tolerance caused by LVH [1]. This suggests that the RER metabolic expenditure differences observed in *Mybpc3^−/−^* mice are not due to differences in physical activity levels but rather an inherent systemic metabolic shift due to cardiac hypertrophy.

We believe that the presence of LVH in *Mybpc3^−/−^* mice caused the metabolic reprogramming observed in metabolically active tissues highly dependent on FAO-OXPHOS. Our histology and functional mitochondrial studies showed similar shifts in metabolic processes. Although it is possible that reduced oxygen availability may contribute to this metabolic shift, our data also suggest that supplementation of exogenous FFAs reversed this phenotype in myocardium (Figure 10E), indicating that lipid utilization and storage could be a key modulator of systemic metabolism in LVH. Previous studies have also provided evidence that increasing the levels of carnitine palmitoyl transporters (CPTs) as a therapeutic target increases fatty acid uptake by the mitochondria, thereby increasing LCFA oxidation and reducing cardiac lipotoxicity in patients with various causes of LVH [12,16,33,34,35]. Another proposed mitochondrial target, α-ketoglutarate dehydrogenase (KGDH), synthesizes succinyl-CoA from α-ketoglutarate as one of the NADH-generating steps of the tricarboxylic acid (TCA) cycle [36,37,38]. Since this reaction is NAD^+^-dependent, KGDH acts as a mitochondrial redox sensor to reversibly suppress mitochondrial activity under extreme oxidative stress [37]. More recently, KGDH has been shown to play a role in facilitating H_2_O_2_ elimination in response to CI blockades in the heart [36]. Therefore, it is possible for cardiomyocytes to generate ATP through OXPHOS, with the right substrates. KGDH expression levels, stability, and activity studies are necessary to determine whether exogenous supplementation of LCFAs counteracts mitochondrial suppression via this mechanism.

**Figure 11:**
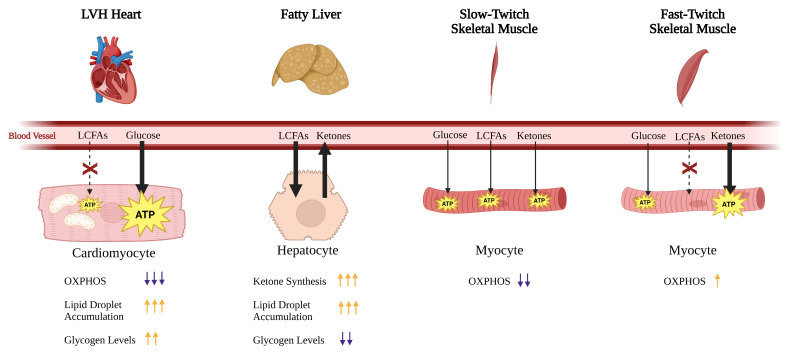
Schematic of the proposed mechanism that supports systemic metabolic rewiring in LVH mice. Loss of FAO OXPHOS in cardiac tissue leads to reduced LCFA uptake and utilization. The compromised cardiomyocytes increase glucose uptake to support energy production via glycolysis and glucose-linked OXPHOS. The liver takes up excess LCFAs for energy production, converting unused acetyl-CoA into ketone bodies to be used by other tissues. Slow-twitch skeletal muscles, which are metabolically reliant on OXPHOS, must metabolically shift to rely mostly on glucose catabolism to drive OXPHOS. Fast-twitch skeletal muscles shift to metabolically rely on ketone bodies generated by the liver. Down arrows (purple) indicate a decreased parameter in LVH contexts. Up arrows (orange) indicate an increased parameter in LVH contexts.

This is corroborated by our in vitro O_2_ consumption data (Figure 7A), which indicate that the altered metabolic state in *Mybpc3^−/−^* mtLV is associated with diminished oxidative capacity, further supporting a shift away from OXPHOS and towards glycolysis (Figure 7B). Interestingly, the provision of exogenous LCFA, both in vitro and in vivo, reversed many metabolic abnormalities observed in *Mybpc3^−/−^* mice, suggesting that their systemic metabolic dysfunction may, in part, be due to impaired lipid utilization outside cardiac tissue. An HFD resulted in loss of hepatic and cardiac lipotoxicity (Figure 9D,E) with increased mitochondrial OXPHOS capacity (Figure 10E) but did not fully rescue the *Mybpc3^−/−^* systemic phenotypes of increased baseline RER (Figure 10A), altered body composition (Figure 9C), or blood glucose and ketone levels indicative of metabolic duress (Figure 10B,C). It is possible that LCFAs supplemented by an HFD may not be utilized by the heart, but instead support FAO in other highly metabolic tissues, such as skeletal muscles. Therefore, we hypothesize that the mechanism underlying shifts in systemic metabolism due to LVH is the hypertrophic heart’s preferential use of most, if not all, bioavailable glucose. We hypothesize that because the liver and skeletal muscles in mice with LVH are able to fuel OXPHOS through FAO, support of cardiac glucose catabolism supersedes glycolytic pathways outside the heart. The specific role of fatty acid, specifically LCFA, uptake and catabolism to acetyl-CoA in the progression of cardiomyopathies and heart failure warrants further investigation in vivo to track fatty acid vs. glucose uptake in the heart, liver, skeletal muscle, brain, and kidney. We suspect that a study like this would show that the *Mybpc3^−/−^* liver is forced to mostly use FAO as its fuel source. Not only are ketone levels constitutively high in *Mybpc3^−/−^* mice—whether glycolytically challenged during a fasting (Figure 5B) or during acute exercise (Figure 6C)—but RER data indicated higher reliance on glucose in the muscle, heart, kidney, liver, and brain tissues. The only way for the body to produce ketones is through liver FAO [39], supporting our hypothesis that compromised heart muscle “forces” a systemic metabolic phenotype; directly affecting liver FAO while indirectly affecting skeletal muscle FAO (Figure 11).

A major limitation of this study was the lack of in vitro female data. We focused on metabolic flux data in one sex because a significantly higher RER moving average was sustained through both light and dark cycles in only male mice. Further, there was no significant difference between body composition in female WT and *Mybpc3^−/−^* mice. We surmised that differences in metabolic flux between the WT and *Mybpc3^−/−^* genotype would be more easily detected in male mice than female mice. Our study using an established mouse model of LVH provides clinically relevant insights into the metabolic consequences of inherited and acquired forms of CVD that result in cardiac hypertrophy. Our observation of systemic metabolic disturbances in *Mybpc3^−/−^* mice due to cardiac hypertrophy may help identify new biomarkers or therapeutic targets that address not only the heart but also the systemic metabolic consequences of cardiac dysfunction. The use of serum acylcarnitine profiling in this study provided insight into the fatty acids primarily not used for heart FAO, then skeletal muscle FAO (Figure 5C–H) [26]. Acylcarnitines are formed inside the mitochondria and then shipped out of the cell when not used as a precursor for acetyl-CoA synthesis via FAO. Since we found a significant increase in several long-chain acylcarnitines in *Mybpc3^−/−^* serum, we can surmise that there is an FAO bottleneck of some sort in heart and/or skeletal muscle.

These profiles are commonly used to screen for inborn errors of metabolism (IEMs) in humans, providing a comprehensive profile of FAO disorders, which cause systemic metabolic dysfunction [40]. Patients with IEMs, particularly those with FAO disorders or hypercholesterolemia, are frequently monitored for the development of CVD [41,42]. Interestingly, we found that the acyl-carnitine profiles of *Mybpc3^−/−^* mice closely match those observed in patients with Short-Chain Acyl-CoA Dehydrogenase Deficiency (SCAHD), a type of IEM characterized by significant increases in C4OH. This similarity supports the possibility that inherited genetic variants that cause hypertrophy in the heart may disrupt systemic metabolism in a manner analogous to IEMs, which have clinical implications in the liver, heart, kidneys, brain, and skeletal muscle [42]. This observation is significant, as it suggests that mutations restricted to cardiac tissue can have wide-ranging effects on metabolic processes throughout the entire organism.

This study provides evidence that LVH caused by loss of the cardiac-specific gene, *Mybpc3*, can indeed induce systemic metabolic dysregulation, extending beyond the heart and affecting whole-body energy balance. This study also provides a valuable framework for studying the systemic metabolic alterations that accompany inherited forms of LVH. The findings underscore the importance of considering the whole-body metabolic effects of cardiac mutations thought to only affect the heart and suggest that therapeutic strategies targeting broader metabolic pathways may be beneficial in managing patients with LVH.

## 4. Materials and Methods

### 4.1. Animals

All animal protocols were approved by Case Western Reserve University’s Institutional Animal Care and Use Committee (protocol #2014-0139; approved 19 September 2023), and all experiments were conducted in accordance with the guidelines and regulations set forth in the Animal Welfare Act (AWA) and PHS Policy on Humane Care and Use of Laboratory Animals. All mice were maintained on a 12 h light/dark cycle in a pathogen-free barrier facility. Male and female *Mybpc3* null (*Mybpc3^−/−^*) mice and SVE129 wild-type controls [7], aged 4–6 months, were used in this study.

### 4.2. Indirect Calorimetry, RER, and Energy Expenditure

Indirect calorimetry (IDC) was used to measure whole-body respiration [43]. This was performed using a Comprehensive Lab Animal Monitoring System (CLAMS; Columbus Instruments, Columbus, OH, USA). Mice were acclimated to the special caging for 12 h prior to a 48 h continuous monitoring period. Mice had ad libitum access to 30 g of pelleted food and drinking water for the duration of the experiment. All O_2_ consumption, CO_2_ release, and respirometry exchange ratio (RER) data were normalized to body mass using CLAX software 2.2 at the time of data collection. Energy expenditure data were generated using total body weight and average O_2_ consumption during the light or dark cycle for each mouse. An ANCOVA test was performed using the JupyterLite Python 3.11.5 environment (jupyter.org).

### 4.3. Assessment of Body Composition

Fat stores were measured to estimate the mouse body composition. Total body weight was measured for each mouse. Mice were then anesthetized with 5% isopropanol followed by cervical dislocation prior to dissection. The gonadal fat pads—the epididymal fat pad (EFP) in males or the peri-ovarian adipose tissue (POAT) in females—were harvested and weighed. The gonadal fat pad to total body weight ratio served as an estimate for body composition.

### 4.4. Histology

A histology study was performed to measure organ-specific energy stores in cardiac and hepatic tissues. Mice were anesthetized with 5% isoflurane. Hearts and liver were perfused with 1× PBS + 1% heparin through the left ventricle, followed by perfusion with 4% paraformaldehyde in 1× PBS. Hearts and livers were isolated and further fixed in 4% paraformaldehyde for an additional 24 h at 4 °C. Samples were embedded in paraffin for Hematoxylin and Eosin (H&E), Masson’s trichrome (MT), and Periodic Acid–Schiff (PAS) staining by the Tissue Resources Core at Case Western Reserve University. Snap-frozen heart and liver sections were fixed in 10% formalin and stained with Oil Red O (ORO) by the Tissue Resources Core at Case Western Reserve University. H&E staining was used to study tissue architecture and cell morphology. MT staining was performed to identify tissue damage due to collagen deposition. PAS staining identified cells with glycogen stores. Slides were scanned at 20× objective with a NanoZoomer (Hamamatsu Photonics, Hamamatsu City, Japan), performed by the Light Microscopy Core at Case Western Reserve University. Heart PAS stains were used to quantify cardiac glycogen stores. Fifteen fields of view were randomly selected from cardiac PAS slides, and glycogen stores were quantified within a 600 μM^2^ gated region. Cardiac and hepatic lipid droplets were counted within a 600 μM^2^ gated region in fifteen random fields of view from heart and liver ORO slides, respectively.

### 4.5. Fasted Glucose and Ketone Measurements

Mice underwent a fasting study to challenge the liver’s ability to regulate in vivo glucose metabolism. Hypoglycemia was induced by placing mice into new cages, singly housed without food for 12 h (overnight). A tail-snip was performed to express a drop of blood, and a hand-held blood glucose and ketone meter (Keto-Mojo, Napa, CA, USA) was used to measure circulating glucose and ketone levels, as previously described [44].

### 4.6. Acylcarnitine Analysis

Circulating fatty acid species that were tagged for mitochondrial transport by the addition of an acyl-CoA group were identified using a clinical mass spectrometry approach. Mice were fasted for 2 h to normalize postprandial glucose spikes prior to blood serum collection from the inferior vena cava. Serum was sent to the Biomedical Genetics Laboratory at Colorado Children’s Hospital (Aurora, CO, USA). The apparatus consisted of a 1260 Infinity II LC system with a 6470 triple quadrupole mass spectrometer (Agilent Technologies, Palo Alto, CA, USA). Chromatographic separation was achieved on an Acquity UPLC BEH C18 Column (130A pore size, 1.7 μm particle size, 2.1 mm inner diameter × 150 mm length; Waters Corporation, Milford, MA, USA) held at 50 °C. The method was modified from that developed by Gucciardi et al. [45]. The mobile phase was a gradient elution of 0.1% formic acid in water (A) to 0.1% formic acid in acetonitrile (B). The flow rate was 0.4 mL/min, and the gradient was as follows: 0 min 1%B, 0.1 min 17%B, 0.28 min 24%B, 0.35 min 26%B, 0.80 min 29%B, 1.71 min 31%B, 2.96 min 34%B, 4.50 min 36%B, 5.44 min 56%B, 6.37 min 70%B, 8.01 min 82%B, 11.30 min 93%B, 12.50 min 95%B, 13.50 min 1%B, and 17 min 1%B. The injection volume was 5 μL, and the total run time was 17 min per sample. The mass spectrometer was equipped with an ESI source operating in positive mode with gas temp 290 °C, gas flow 5 L/min, nebulizer 35 psi, sheath gas temp 400 °C, sheath gas flow 12 L/min, and capillary voltage 3600 V. Data were acquired in multiple reaction monitoring (MRM) mode. Data were acquired using MassHunter Acquisition 9.0 and processed using MassHunter Quantitative Analysis 10.2.

### 4.7. High-Resolution Respirometry in Isolated Mitochondria

Mitochondria isolated from the left ventricular (LV) and skeletal muscle provided insight into the metabolic flexibility within that tissue. LV tissue (~45 mg), quadriceps tissue (~30 mg), or one soleus (~5 mg) was freshly isolated from wild-type or *Mybpc3^−/−^* mice. All tissue was stored and cleaned three times in ice-cold BIOPS buffer containing 10 mM Ca^2+^-EGTA (as prepared according to [46]), 0.1 μM CaCl_2_, 20 mM imidazole, 20 mM taurine, 50 mM K-MES, 0.5 mM dithiothreitol, 6.56 mM MgCl_2_, 5.77 mM ATP, and 15 mM phosphocreatine. The tissue was cleaned of ligaments, weighed, and minced using a razor and a Petri dish on ice. The left ventricle tissue mince was prepared as previously described [47] to produce freshly isolated mitochondria from the left ventricle (mtLV). The skeletal muscle tissue mince was further processed as previously described [47] to produce freshly isolated mitochondria from the quadriceps (mtQuad) and soleus (mtSoleus). Intact mitochondria were resuspended in 20 mL/g of a wet tissue mass MiR05 buffer. An Oroboros Oxygraph-2K (O2k) (Oroboros Instruments, Innsbruck, Austria) was used to assess Complex I (CI), Complex II (CII), and maximal respiration of isolated mitochondria. A total of 20 μL of freshly isolated mitochondria (approximately 30 μg) was added to 2 mL of O_2_-equilibrated MiR05 buffer in the O2k chambers. Once the sample baseline was stable, cytochrome C (0.01 mM) was added to the chamber to assess mitochondrial outer membrane integrity. Malate (1.0 mM), ADP (1.25 mM), pyruvate (5.0 mM), glutamate (5.0 mM), and succinate (10 mM) were used to stimulate Complex I and II respiration. Carbonyl cyanide m-chlorophenyl hydrazone (CCCP, 1.0 μM) was added to uncouple the electron transport chain (ETC) and measure maximal oxidation. Finally, rotenone (0.5 μM) and antimycin a (2.5 μM) were added to stop mitochondrial respiration.

### 4.8. High-Resolution Respirometry of Permeabilized Muscle Fibers

Skeletal muscles—quadriceps and soleus—were isolated from WT and *Mybpc3^−/−^* mice. Each tissue was washed in ice-cold BIOPS buffer, and then the fibers were teased apart using ultra-fine-tip forceps. Tissues were then permeabilized with 5 mg/mL saponin in BIOPS for 20 min. An Oroboros Oxygraph-2K (O2k) (Oroboros Instruments, Innsbruck, Austria) was used to assess Complex I, Complex II, and maximal respiration of permeabilized tissue. A total of 3–5 mg of tissue was added to 2 mL of an O_2_-equilibrated MiR05 buffer in the O2k chambers. Once the sample baseline was stable, palmitoyl-L-carnitine (0.04 mM) was added to the chamber to supply the tissue with a long-chain fatty acid to measure FAO OXPHOS. Malate (0.5 mM), glutamate (10 mM), pyruvate (5 mM), ADP (2.5 mM), cytochrome c (10 μM), and succinate (10 mM) were added to measure Complex I and II respiration. CCCP (1.0 μM) was added to uncouple the ETC from ATP synthase and measure maximal oxidation. Finally, rotenone (0.5 μM) and antimycin a (2.5 μM) were added to stop mitochondrial respiration.

### 4.9. Exercise Challenge

To challenge glycolytic capacity and de novo glucose synthesis under metabolic stress, mice were run to exhaustion on a single-lane isolated rodent treadmill (Columbus Instruments, Columbus, OH, USA). Exercise-naïve mice were acclimated for 10 min with the belt off and foot shockers on (40 Hz). Data collection began after the acclimation period. Mice walked on the treadmill at 5 m/min, 0% grade, for 2.5 min. The treadmill grade was then increased to 25% for 2.5 min at a speed of 5 m/min. The speed was increased from 5 m/min to 14 m/min over the course of 3 min, and then held at 14 m/min until exhaustion. All data were collected at 8 AM. Mice had access to food and water before the exercise challenge.

## Figures and Tables

**Table 1:** Two-way ANCOVA analysis was performed, assuming that body mass was a covariate of O_2_ consumption. Genotype, sex, and genotype * sex had no significant effect on O_2_ consumption.

*Mybpc3^−/−^* vs. Wild-Type ANCOVA Analysis								
	Light Cycle				Dark Cycle			
	Sum of Squares	DF	F	*p*-Value	Sum of Squares	DF	F	*p*-Value
Genotype	3.97 × 10^4^	1.0	0.0862	0.7712	1.46 × 10^4^	1.0	0.0284	0.8674
Sex	6.44 × 10^4^	1.0	0.1400	0.7111	3.20 × 10^5^	1.0	0.6239	0.4365
Genotype * Sex	1.80 × 10^5^	1.0	0.3829	0.5412	1.16 × 10^5^	1.0	0.2264	0.6380
Body Mass	5.29 × 10^5^	1.0	1.1255	0.2981	8.88 × 10^5^	1.0	1.7344	0.1989
Residual	1.29 × 10^7^	27.0	NA	NA	1.38 × 10^7^	27.0	NA	NA

**Table 2:** One-way ANCOVA analysis was performed assuming that body mass was a covariate of O_2_ consumption. Genotype had no significant effect on O_2_ consumption.

HFD *Mybpc3^−/−^* vs. Wild-Type ANCOVA Analysis				
	Sum of Squares	df	F	*p*-Value
Genotype	8.95 × 10^4^	1.0	0.9538	0.3543
Body Mass	1.22 × 10^5^	1.0	1.3014	0.2834
Residual	8.45 × 10^5^	27.0	NA	NA

## Data Availability

The original contributions presented in this study are included in the article/Supplementary Materials. Further inquiries can be directed to the corresponding authors.

## Supplementary Materials

The following supporting information can be downloaded at https://www.mdpi.com/article/10.3390/ijms262010111/s1.
