# Supplementary material for: Systemic Metabolic Rewiring in a Mouse Model of Left Ventricular Hypertrophy
Source: Int J Mol Sci. 2025 Oct 17;26(20):10111. doi: 10.3390/ijms262010111 (PMC12562477; doi:10.3390/ijms262010111)
Supplement: Supplementary file 1 [file ijms-26-10111-s001.zip › ijms-3885454-supplementary.pdf]

Supplementary Material

1 Supplementary Data

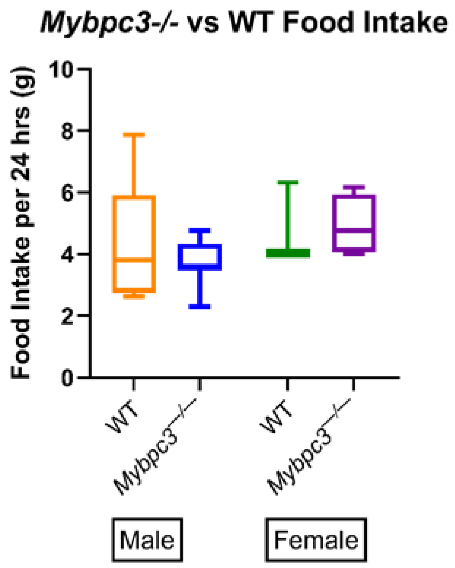

Figure S1. Food intake data are represented as a 24 hr average, measured over the course of 24 hrs. There was no significant difference between male *Mybpc3*$^{-/-}$ (N=7) and WT males (N=6; P=0.4515). There was no significant difference between female *Mybpc3*$^{-/-}$ mice (N=4) and WT mice (N=3; P=0.8731). Error bars represent SD.

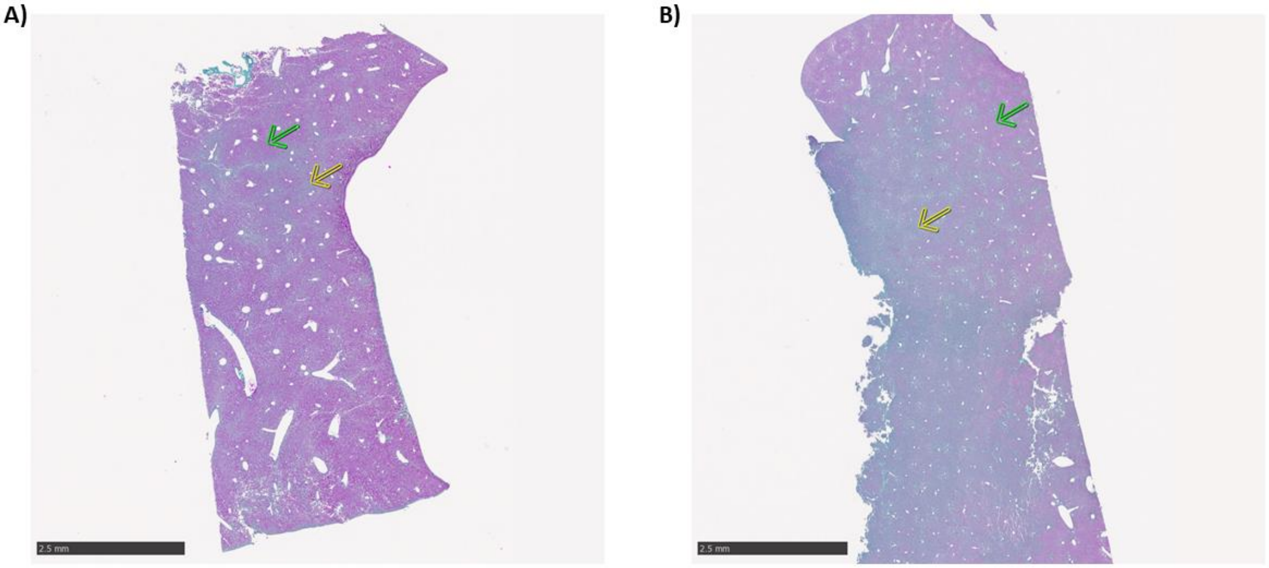

Figure S2. (A) whole-slide scan of liver stained with PAS isolated from a WT mouse. The green arrow indicates an example of a glycogen-rich region in the liver. The yellow arrow indicates a glycogen-deplete region in the liver. (**B)** Whole-slide scan of liver section stained with PAS isolated from a *Mybpc3*$^{-/-}$ mouse. The green arrow indicates an example of a glycogen-rich region in the liver. The yellow arrow indicates a glycogen-deplete region in the liver.

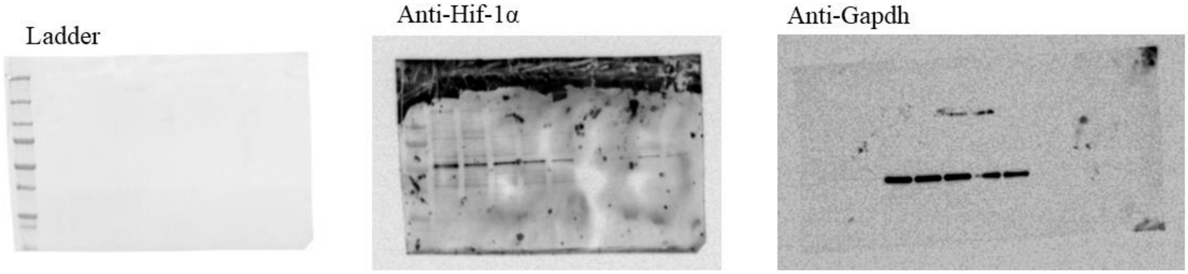

Figure S3. Full membrane images of anti-Hif-1α, anti-Gapdh, and ladder immunoblots.

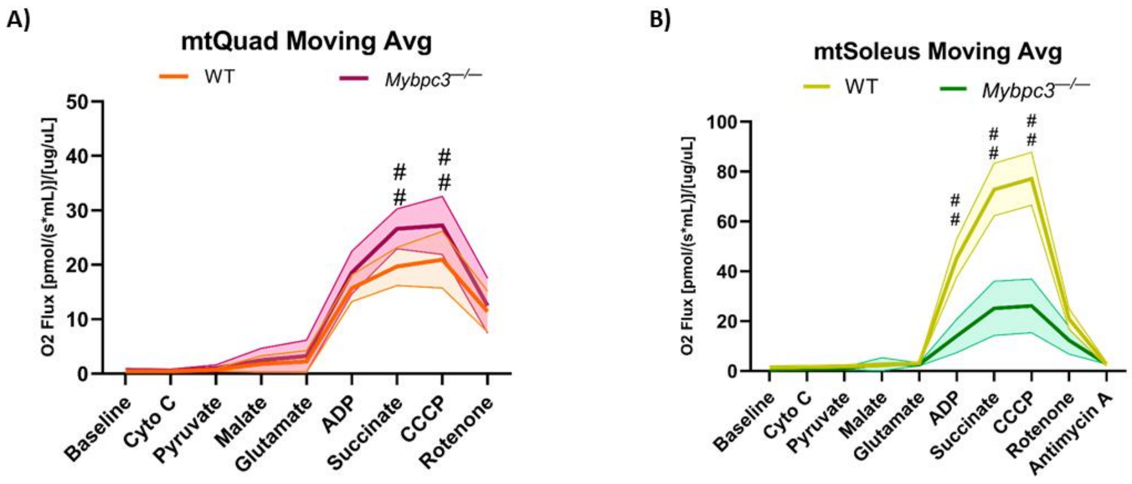

Figure S4. (A) Oxygen consumption of mtQuad in response to various substrates was plotted as a moving mean. Multiple t-test analysis showed *Mybpc3*$^{-/-}$ mtQuad (N=6) had significantly higher oxygen consumption with treatment of succinate (Q=0.001188) and CCCP (Q=.0001890) compared to WT mtQuad (N=6). **B)** Oxygen consumption of mtSoleus in response to various substrates was plotted as a moving mean. Multiple t-test analysis showed *Mybpc3*$^{-/-}$ mtSoleus (N=6) had significantly higher oxygen consumption with treatment of ADP (Q=0.000288), Succinate (Q=0.000288), and CCCP (Q=0.000288) compared to WT mice (N=6). Error bars (shaded region) represents SD; ## = Q<0.001.

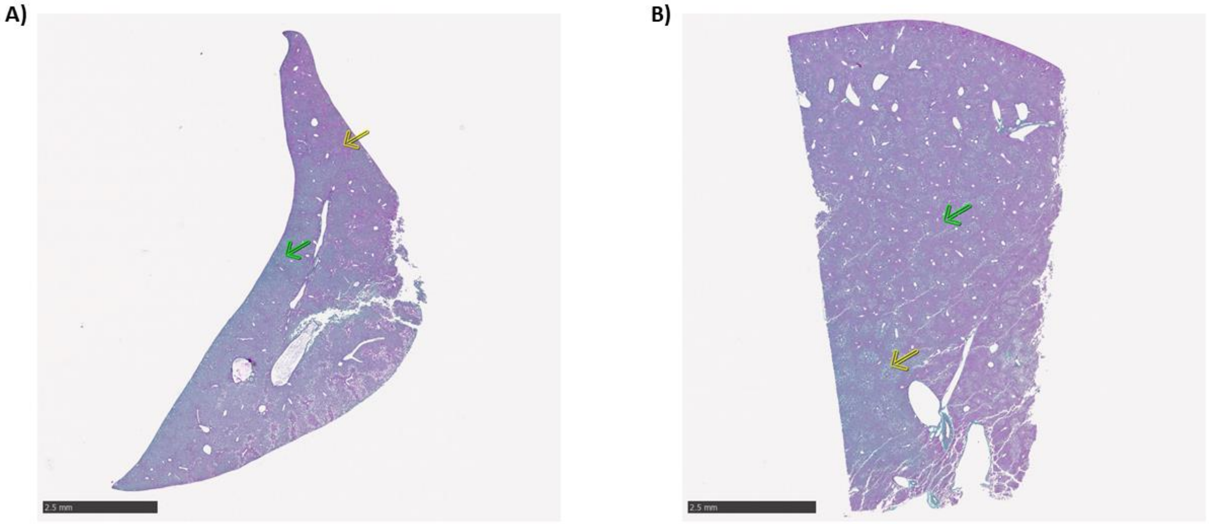

Figure S5. *Mybpc3*$^{-/-}$ and WT mice were fed a HFD for 10 weeks. (**A**) whole-slide scan of liver stained with PAS isolated from a WT mouse. The green arrow indicates an example of a glycogen-rich region in the liver. The yellow arrow indicates a glycogen-deplete region in the liver. (**B**) Whole-slide scan of liver section stained with PAS isolated from a *Mybpc3*$^{-/-}$ mouse. The green arrow indicates an example of a glycogen-rich region in the liver. The yellow arrow indicates a glycogen-deplete region in the liver.

2 Supplementary Table

2.1 **Table S1.** Quantification and statistical analysis of circulating acylcarnitine concentrations in *Mybpc3*$^{-/-}$ vs WT mice.

***Mybpc3-/-* vs Wild-type Acylcarnitine Profiling**

	Wild-type				Mybpc3-/-					
**Substrate Name**	**Final Concentration**			**WT Avg**	**Final Concentration**			**Mybpc3-/- Avg**	**P-value**	**FC (Mybpc3-/- / WT)**
C0	19.7816	25.7394	18.5903	21.3704	24.2841	16.8109	19.4745	20.1898	0.7235	0.94
C2	18.7453	30.0778	21.6525	23.4919	25.6996	22.2359	22.7533	23.5629	0.9850	1.00
C3	0.7393	1.0653	0.8966	0.9004	0.9667	0.7619	0.8212	0.8499	0.6758	0.94
C4	0.4153	0.4718	0.3996	0.4289	0.6492	0.3348	0.3526	0.4455	0.8810	1.04
C4OH	0.1561	0.1467	0.1044	0.1357	0.2764	0.2225	0.2282	0.2424	0.0103	1.79
C5	0.1270	0.2717	0.2116	0.2034	0.3232	0.2011	0.1939	0.2394	0.5772	1.18
C5_isomers	0.0893	0.1449	0.0935	0.1092	0.1024	0.0738	0.0762	0.0841	0.2795	0.77
C5:1	0.0048	0.0074	0.0064	0.0062	0.0125	0.0081	0.0070	0.0092	0.1789	1.48
C5OH	0.0000	0.0717	0.0568	0.0428	0.0851	0.0592	0.0612	0.0685	0.3338	1.60
C6	0.0596	0.1175	0.0902	0.0891	0.1108	0.1100	0.0890	0.1033	0.4794	1.16
C6OH	0.0081	0.0095	0.0078	0.0085	0.0107	0.0104	0.0110	0.0107	0.0155	1.26
C8	0.0211	0.0161	0.0105	0.0159	0.0130	0.0129	0.0072	0.0110	0.2491	0.69
C8:1	0.0086	0.0083	0.0059	0.0076	0.0042	0.0045	0.0026	0.0038	0.0210	0.50
C8OH	0.0133	0.0143	0.0120	0.0132	0.0145	0.0129	0.0116	0.0130	0.8609	0.98
C10	0.0000	0.0000	0.0000	0.0000	0.0000	0.0000	0.0000	0.0000	NA	NA
C10:1	0.0000	0.0000	0.0000	0.0000	0.0000	0.0000	0.0000	0.0000	NA	NA
C10:2	0.0000	0.0000	0.0000	0.0000	0.0000	0.0000	0.0000	0.0000	NA	NA
C10OH	0.0087	0.0102	0.0063	0.0084	0.0058	0.0061	0.0037	0.0052	0.0788	0.62
C12	0.0127	0.0203	0.0075	0.0135	0.0120	0.0070	0.0034	0.0075	0.2489	0.55
C12:1	0.0118	0.0142	0.0095	0.0118	0.0107	0.0127	0.0063	0.0099	0.4528	0.84
C12OH	0.0000	0.0000	0.0000	0.0000	0.0000	0.0000	0.0000	0.0000	NA	NA
C14	0.0411	0.1010	0.0646	0.0689	0.0711	0.0620	0.0668	0.0666	0.9039	0.97
C14:1	0.0737	0.0929	0.0524	0.0730	0.1095	0.0763	0.0646	0.0835	0.5886	1.14
C14:1OH	0.0000	0.0000	0.0000	0.0000	0.0000	0.0000	0.0000	0.0000	NA	NA
C14:2	0.0262	0.0392	0.0242	0.0299	0.0308	0.0237	0.0171	0.0239	0.3841	0.80
C14OH	0.0000	0.0000	0.0000	0.0000	0.0000	0.0000	0.0000	0.0000	NA	NA
C16	0.0401	0.1127	0.0747	0.0758	0.1138	0.1063	0.1342	0.1181	0.1343	1.56
C16:1	0.0266	0.0524	0.0301	0.0364	0.0693	0.0569	0.0419	0.0560	0.1572	1.54
C16:1OH	0.0033	0.0050	0.0038	0.0040	0.0064	0.0060	0.0067	0.0064	0.0127	1.58
C16OH	0.0069	0.0134	0.0093	0.0099	0.0161	0.0125	0.0147	0.0144	0.1029	1.46
C18	0.0738	0.0531	0.0247	0.0505	0.0864	0.0503	0.1080	0.0816	0.2319	1.61
C18:1	0.0173	0.0468	0.0219	0.0287	0.1014	0.0634	0.0808	0.0819	0.0205	2.86
C18:1OH	0.0418	0.0570	0.0419	0.0469	0.0389	0.0477	0.0469	0.0445	0.6992	0.95
C18:2	0.0819	0.0955	0.0729	0.0834	0.1380	0.0849	0.1180	0.1136	0.1470	1.36
C18:2OH	0.0235	0.0418	0.0277	0.0310	0.0465	0.0320	0.0357	0.0381	0.3722	1.23
C18OH	0.0000	0.0000	0.0000	0.0000	0.0000	0.0000	0.0000	0.0000	NA	NA
